# Effect of surfactant concentration on the evaporation-driven deposition of carbon nanotubes: from coffee-ring effect to strain sensing†

**DOI:** 10.1039/d2ra03833a

**Published:** 2022-11-07

**Authors:** Mohammad Jalal Inanlu, Jafar Farhadi, Ehsan Ansari, Saina Charkas, Vahid Bazargan

**Affiliations:** School of Mechanical Engineering, College of Engineering, University of Tehran Tehran Iran jafarfarhadi@ut.ac.ir vbazargan@ut.ac.ir; School of Electrical and Computer Engineering, College of Engineering, University of Tehran Tehran Iran

## Abstract

Carbon nanotubes (CNTs) as electrically conductive materials are of great importance in the fabrication of flexible electronic devices and wearable sensors. In this regard, the evaporation-driven self-assembly of CNTs has attracted increasing attention. CNT-based applications are mostly concerned with the alignment of CNTs and the density of CNT films. In the present work, we focus on the latter by trying to achieve an optimal evaporation-driven deposition with the densest CNT ring. Although surfactants are used for effective dispersion and colloidal stabilization of CNTs in the aqueous phase, their excessive usage induces Marangoni eddies in the evaporating sessile droplets, leading to poor ring depositions. Thus, there is an optimum surfactant concentration that contributes to CNTs deagglomeration and results in the densest ring-like deposition with relatively high thickness. We report that this optimum concentration for sodium dodecyl sulfate (SDS) as a surfactant can be approximately considered as much as the concentration of multi-walled carbon nanotubes (MWCNTs) as the colloidal nanoparticles. Optimal depositions show the lowest electrical resistances for each CNT concentration, making them suitable for electronic applications. We also propose the multiple depositions method in which a new droplet is printed after the complete evaporation of the previous droplet. This method can lead to denser rings with a higher conductivity using lower concentrations of CNTs. Lastly, we fabricate strain sensors based on the optimal evaporation-driven deposition of CNTs which show higher gauge factors than the commercial strain gauges, corroborating the applicability of our method.

## Abstract 2

Our discovery reveals the existence of an optimum SDS/CNT concentration ratio to achieve the highest conductivity of ring deposition for strain sensing.

## Graphical abstract

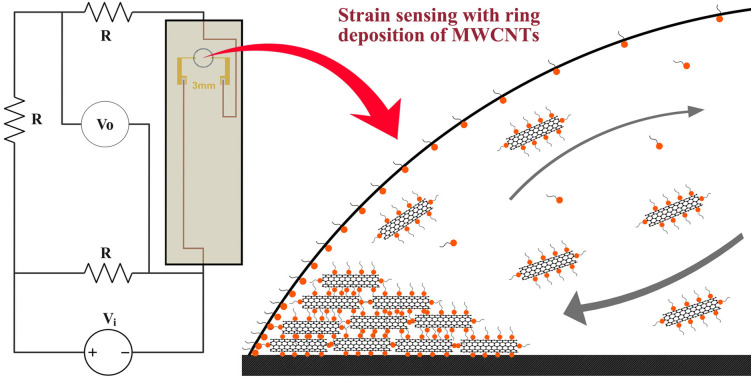

## Introduction

Carbon nanotubes (CNTs) have recently attracted great interest in many industries due to their excellent electrical and mechanical properties. As one of the most important applications, CNTs are promising candidates for fabricating high-performance flexible integrated circuits^1^ due to their great mechanical flexibility, high carrier mobility and current carrying capacity, high length-to-diameter aspect ratio for effective electrostatic control, and solution-processability for low-cost production.^2^ In addition to the inkjet printing of CNTs for flexible electronics, they are also exploited in fabrication of transparent conducting films,^3,4^ wearable devices for healthcare monitoring,^5^ chemical sensors^6^ and various sensing devices including temperature,^7,8^ pH,^9^ pressure,^10^ and novel CNT-based strain sensors.^11^ Having high values of strain sensitivity, also called gauge factor (which is defined as the ratio of relative change in electrical resistance to the mechanical strain – piezoresistive effect), makes CNTs great candidates for strain sensing.^12^ In such applications, the coffee-ring effect plays the leading role in the self-assembly of CNTs.^13^ Coffee-ring phenomenon is observed when a particle-laden droplet dries on a solid substrate and leaves a ring-like deposit of particles along the perimeter of the original droplet as a result of evaporation-induced flow.^14^ Considering contact line dynamics during evaporation of particle-laden droplets, smart patterns and ordered microstructures can be printed on the surface.^15^

Both chemical and physical approaches are used to improve the dispersity and stability of CNTs in the aqueous phase.^16^ The chemical approaches, such as covalent treatment with acids, may cause structural damage to the CNTs. On the other hand, non-covalent treatment with the help of dispersing agents does not suffer from those disadvantages. As a physical approach, surfactant addition is preferred due to the easier removal in comparison with other dispersing agents. Surfactants can disperse CNTs in an aqueous solution by means of specific interactions. The hydrophobic tail of the surfactant molecule adsorbs on the surface of CNT bundles, while the hydrophilic head associates with water for dissolution.^17^ It should be noted that in evaporation-driven approaches for self-assembly of CNTs, high concentrations of surfactant lead to attenuated ring/strip^18^ or even uniform deposition/film^19^ of CNTs. This behavior is due to the surfactant-induced Marangoni eddies, suppressing the capillary flow that drives ring deposition in the first place.^20,21^ Therefore, ink formulation has a non-negligible impact on the quality of inkjet-printed CNT networks.^22^ In a specified formulation, increasing the CNT concentration promotes the pinning of the contact line and consequently enhances the ring density.^23^ Although uniform deposition is often desired in surface coating applications, a controllable concentrated deposition pattern is of interest in applications associated with the electrical conductivity of deposited CNTs.^24^ It should also be noted that although a higher concentration of ionic surfactants can lead to a higher solution conductivity due to the increase in the concentration of free ions,^25^ it cannot alter the conductivity of dried deposition of colloidal CNTs significantly.^13^

There are mainly two concerns corresponding to the self-assembly of CNTs,^26^ one is the density of the deposit^27^ that has been elaborated on briefly, and the other one is the high degree of CNTs alignment.^28^ A set of approaches including post-synthesis and *in situ* growth methods has been proposed to align CNTs.^29^ Evaporation-driven (or in general flow-directed) alignment as one of the post-synthesis approaches can yield a feasible process with nanometer precision for self-assembly of CNTs.^30^ It was shown that the capillary flow inside an evaporating droplet deposits CNTs along the contact line with a high degree of alignment.^31^ By controlling the evaporation-induced flow, Sharma *et al.*^32^ reported a fine alignment of CNTs alongside the cylindrical microdroplets with a resolution of nanometer for CNTs positioning. They were also able to align CNTs along the centerline of the cylindrical nanodroplets.^33^ Following their pioneering efforts, numerous works have been carried out to align CNTs using inkjet printing based on the coffee-ring effect.^34,35^

Different methods are recommended for the fabrication of CNT-based strain sensors. By inkjet printing of CNT solutions, Michelis *et al.*^36^ were able to fabricate a flexible strain sensor. Their ohmic sensor provided a hysteresis-free strain gauge with low variability in resistance. Another fully inkjet-printed CNT-based strain sensor was fabricated by Kao and coworkers.^37^ They reported that multi-pass printing leads to a higher gauge factor due to the higher density and uniformity of CNTs. The higher the gauge factor, the more sensitive the strain gauge is to applied stress. Li *et al.*^38^ fabricated a strain sensor employing capillary-assisted deposition of CNT films on polymeric surfaces with micropatterned structures and reported as the CNT concentration increases, the film resistance decreases, and reaching a constant gauge factor becomes attainable. They argued that the evaporation-driven self-assembly of CNTs would find applications in flexible electronics. Although they tried to restrain the coffee-ring effect, we exploit this well-known phenomenon in sessile water droplets to deposit a dense CNT ring with relatively high conductivity and fabricate a novel strain sensor. Using water as a non-toxic solvent allows nanoparticles to deposit gently at the contact line and form an entangled CNT network.^39^

What has been neglected in previous works using the self-assembly method is the effect of surfactant concentration on several aspects including colloidal stability of CNT suspension, flow inside the evaporating CNT-laden droplet, the density of the deposited ring, and electrical behavior of the CNT ring. Using multi-walled carbon nanotubes (MWCNTs) as the colloidal nanoparticles and sodium dodecyl sulfate (SDS) as the dispersing agent, the present effort aims to find the optimum SDS/MWCNT concentration ratio by altering both concentrations of MWCNT and SDS. For a constant amount of MWCNTs (nominal concentration), this optimum ratio results in the densest ring with the highest electrical conductivity achieved by evaporation-driven positioning. Even though single-walled carbon nanotubes (SWCNTs) tend to align better than MWCNTs due to their higher flexibility,^30,31^ MWCNTs have shown great potential in applications with a major focus on the structural density of closely packed CNT networks.^40^ Considering the purpose of this study and the relatively lower cost of MWCNTs, they were selected for carrying out all the experiments. Hereafter, we use CNT/CNTs instead of MWCNT/MWCNTs for more convenience. Also, SDS surfactant was chosen due to its effortless processing procedures and wide use in CNT-based applications. For the first time, to the best of our knowledge, we attempt the multiple depositions method to improve the ring density in a constant nominal concentration. Lastly, to evaluate the applicability of the method, we try fabricating a simple CNT-based strain sensor that appears to be even better than the commercial ones despite using an inexpensive method.

## Material and methods

### Suspension preparation

Proper amounts of MWCNTs (20–30 nm, purity >95%) purchased from US Research Nanomaterials, Inc. and SDS purchased from Merck were weighted using an A&D HR-200 Analytical Balance (readability: 0.1 mg). After the addition of deionized water, the suspensions were sonicated in an ultrasonic bath sonicator (Sigma) for 10 minutes with 80% power to ensure that CNTs are effectively separated from each other to get homogeneous colloidal suspensions and to ensure that enough SDS surfactants are adsorbed to the surface of CNTs, so they would not be agglomerated later. In order to remove undispersed agglomerates of CNTs in the colloidal suspensions, they were centrifuged for 10 minutes at 3600 rpm. The suspensions were prepared in the volume of 40 mL for four nominal CNT concentrations (*ϕ*), namely, 0.05, 0.1, 0.2, and 0.4 wt% in different SDS concentrations (*ψ*), namely, 0.025, 0.05, 0.1, 0.2, 0.4, and 0.8 wt%. The range of the SDS concentration was changed for each suspension to be in the range of the CNT concentration since as the CNT concentration increases in the colloidal suspension, more SDS is needed for the dispersion of the CNTs. It should be noted that the true concentrations of CNTs may be different from the nominal values, as they are listed in Table S1.† However, the nominal concentrations are still reported in order to make comparison possible and simplify the discussion of the results.

### Experimental procedure

Proceeding the suspension preparation, a droplet of 2 μL for each case was cast on a glass substrate using a micropipette, and images were taken of the final deposition after the complete evaporation using a Basler ace acA800-510uc camera mounted on a Leitz HM-LUX microscope. The videos of the ESI† were also taken with the same camera and microscope, but with a higher magnification lens. For the multiple depositions part, since the deposition was repeated several times, in order to avoid the droplets being protruded from the original ring, very accurate control over the deposition was required, and instead of using a micropipette, a Jikan CAG-20 PE goniometer was used for the injection of the suspension which uses accurate servomotors to control the injection rate and stage height. After injection of the first droplet, the stage was lowered and after complete evaporation of the droplet, it was raised again to print the second droplet and the procedure was repeated as much as needed for each case. The complete evaporation condition was confirmed when all the solvent (water) vanished. The ratio of the ring width (*w*) of the deposits to their final deposition radius (*r*) was calculated using two image processing codes in MATLAB that detect these values based on the RGB difference due to the contrast between the substrate and the deposited CNTs. As for getting the *r* value, the total area of the deposition was calculated and equalized to π*r*^2^. To measure the electrical resistance (*R*) of the rings, a customized Keithley 236 Sourcemeter was used with an added Karl Suss PM5 probe station with gold contacts. The probes were placed at two ends of the diameter of the concentric circle, with the radius equal to the average value of the inner and outer radii of the ring. In order to be sure about the long-term electrical conductivity of the CNT rings, electrical resistance measurements were conducted 7–10 days after depositing droplets. All of these procedures were repeated 10 times for ring width analysis and 5 times for electrical resistance measurement to reduce the uncertainties and increase the confidence of the measurements. So the values are reported as the mean values along with their standard deviations. The deposited CNTs were coated with a layer of 5 nm gold using DC magnetron sputtering technique and scanning electron microscopy (SEM) images were taken using a Hitachi S4160 field emission scanning electron microscope under an accelerating voltage of 20 kV.

### Strain gauge fabrication

**Fig. 1:**
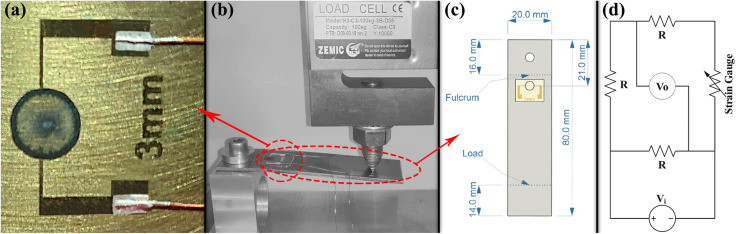
(a) Ring deposition of CNTs on the metal patterned PET sheet, (b) mechanical force setup along with the fabricated strain gauge, (c) dimensions of the cantilever beam, and (d) the circuit designed for strain sensing.

Two groups of CNT strain gauges were fabricated by using two different colloidal suspensions, namely, *ϕ* = *ψ* = 0.2 wt% and *ϕ* = *ψ* = 0.4 wt%, which had the best electrical conductivity as shown in the next section. Each group consists of five similar samples with the same colloid. The electrical resistances of the corresponding CNT rings were 39.7 ± 0.2 kΩ and 12.4 ± 0.1 kΩ, respectively. Any discrepancies between these values and the ones reported in the next section are due to the differences in fabrication methods. CNT strain gauges were fabricated on 200 μm thick transparent polyethylene terephthalate (PET) sheets. For each sample, a Cr/Au (20 nm/200 nm) metal layer was deposited on a separate PET sheet using a VAS OF-1807 RF-sputtering physical vapor deposition system. The sputtering depositions of Cr and Au were performed in an Ar gas ambient with a pressure of 15 mTorr and plasma power of 200 W for 1.5 and 10 minutes, respectively. Then, using a standard lithography method, the metal layer was patterned for all samples as shown in Fig. 1a. The lithography process started with spin-coating of Shipley 1813 photoresist on the surface and continued by exposing samples to UV using a Karl Suss MA6/BA6 (Suss MicroTec, Germany) mask aligner system, and then patterns were developed by MICROPOSIT 351 solution and etched witch Au and Cr etchants. Finally, samples were rinsed with acetone and deionized water. The pattern includes two 0.2 mm arms that were used as bottom-contact electrodes for the deposited CNT rings.

For the electromechanical characterization of the strain gauges, they were installed on similar cantilever beams made of 304 stainless steel with a thickness of 1.5 mm as shown in Fig. 1b and c. Before installing strain gauges, the steel beams' surface preparation process was done in two steps. The surface was roughened with emery paper grade 180 to enhance the contact surface and bonding force. Then the installation area was degreased and cleaned with acetone, isopropyl alcohol, and deionized water, respectively. Using a cyanoacrylate glue (HBM Z70 strain gauge adhesive), metal patterned PET sheets were installed on the steel beams. The schematic of the measurement setup is shown in Fig. 1c. Then using Jikan CAG-20 PE goniometer, a droplet of 2 μL of the mentioned suspensions was printed at the center of the two gold electrode arms for each device and gave the droplet 30 minutes to be evaporated and form the ring deposition. The deposited CNT rings were annealed for 10 minutes at 50 °C in the ambient pressure to stabilize the resistance of the fabricated CNT strain gauges. Although a small amount of moisture might still remain in the rings, the results' consistency would not be altered, as can be confirmed by the statistical significance analysis of the results. 0.5 mm lacquered copper wires were used for electrical connections between the circuits and strain sensors. The wires were soldered to the Wheatstone bridge circuits and glued to the metal contacts of CNT rings with silver paste. Steel cantilever beams on which strain gauges were installed were loaded from 0 to 80 N in 8 steps before reaching their yield points. Mechanical force setup is illustrated in Fig. 1b. As a reference gauge, a commercial strain sensor (HBM type 1.5/120LG11) with the electrical resistance of 120 Ω was installed on another similar cantilever beam and loaded similarly. Since the reference gauge and CNT rings had different electrical resistances, different Wheatstone bridges were designed, one for each sample (including the reference gauge). In each Wheatstone bridge, the electrical resistance of each resistor was equal to the gauge resistance. Fig. 1d illustrates the schematic of the Wheatstone bridge and designed circuit. For all samples, a 5.1 V electrical potential was used to supply Wheatstone bridges, and the electrical readout is performed using a Keithley 236 measurement unit at each loading step.

## Results and discussion

### Single deposition

**Fig. 2:**
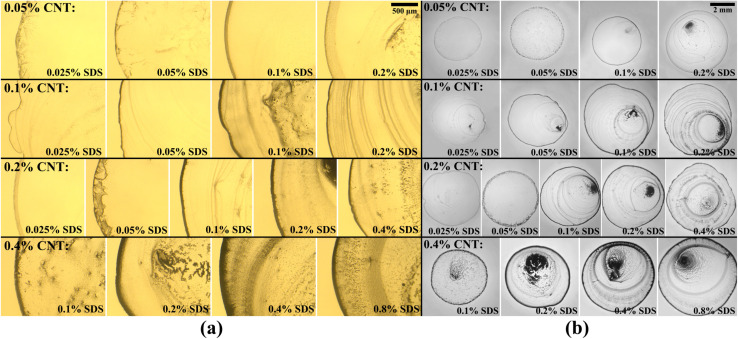
Final deposition patterns of dried colloidal droplets for different CNT and SDS concentrations in (a) close view of the contact line and (b) full view of the droplet. One can notice the change in ring width and deposition radius as SDS concentration increases for each CNT concentration. The corresponding deposits from close view and full view are not necessarily from the same droplet.

**Fig. 3:**
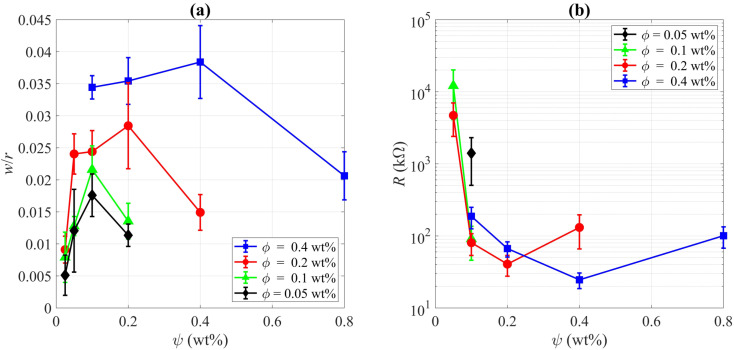
(a) The ratio of the ring width to the deposition radius and (b) the electrical resistance of the deposited CNT rings for different CNT concentrations (*ϕ*) *versus* different SDS concentrations (*ψ*). One can notice that at a specific SDS concentration, the dimensionless ring width is the highest and the corresponding electrical resistance is the lowest for each CNT concentration. Please note that the electrical resistance results are not shown for the rings with sparse CNTs due to high measurement errors (three samples for *ϕ* = 0.05 wt%, two samples for *ϕ* = 0.1 wt%, and one sample for *ϕ* = 0.2 wt%). The conductivity would be practically considered zero for them (*R* → ∞).

Final deposition patterns of dried colloidal droplets for different CNT concentrations in different SDS concentrations are depicted in Fig. 2. Checking Fig. 2a for each CNT concentration (from left to right in each row), it can be seen that as the surfactant concentration increases, the width of the deposited ring of CNTs increases to some point, and then it decreases. This can also be seen in Fig. 3a that for each CNT concentration, there is a specific SDS concentration at which the dimensionless width of the ring deposition (*w*/*r*) is maximum and the densest CNT ring is observed. It is also deduced that as the CNT concentration of the colloids increases, this ratio is generally increased, but more importantly, the SDS concentration at which we get the densest ring also increases. Aside from the change in the density of the CNT ring, it is understood from Fig. 2b that as the surfactant concentration is increased, the radius of the final deposit also increases. That is simply due to the decrease in the surface tension of the colloid as a result of surfactant addition. The decrease in surface tension will contribute to a lower contact angle, which leads to a higher wettability for a fixed volume. The surface tension values were measured and listed in Table S2.† The electrical resistances of these rings were also measured, and the results are depicted in Fig. 3b. Comparing Fig. 3a and b, it is readily apparent that the denser the CNT ring, the higher the electrical conductivity (the lower the resistance). Comparing the results of different concentrations in Fig. 3b, it is noticeable that with the increase in CNT concentration, the electrical conductivity will be improved. However, the surfactant-assisted conductivity improvement is much more noticeable at lower CNT concentrations compared to higher concentrations (as can be perceived from the steeper slope of the lines in lower CNT concentrations). Thus, there is a trade-off between the desired electrical conductivity and the additional amount of CNTs that must be used. It should also be noted that in cases where the rings were not thick enough, the corresponding electrical resistances were measured with relatively high errors. In more intensified conditions, the ring resistance cannot be measured correctly, and the conductivity would be practically considered zero. So the results related to the rings with sparse CNTs are not shown in Fig. 3b. Once more, putting Fig. 3a and b in perspective, it is understood that there is a specific surfactant concentration at which the densest ring with the highest electrical conductivity is achieved.

**Fig. 4:**
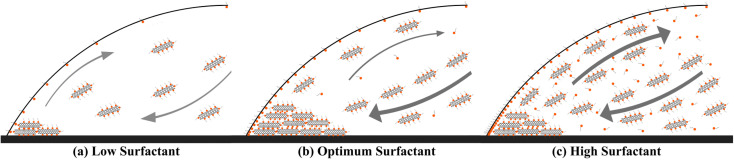
Schematic representation of the capillary and Marangoni flows in evaporating CNT-laden droplets with (a) low, (b) optimum, and (c) high surfactant concentrations. For low concentrations of SDS, there are not enough stabilized dispersed CNTs to form a dense ring, and for high concentrations of SDS, the Marangoni flow gets so strong that the inward capillary flow is repressed. At the optimum concentration of SDS, the best ring with the highest density and conductivity is observed.

This behavior can be explained based on the effect of added surfactant (for the colloidal stability) on the physics of the fluid inside the droplet, which has been majorly ignored in the literature. In our previous work, we found that in a surfactant-laden colloidal droplet, there would be a strong interaction between nanoparticles and surfactants.^20^ So not only the surfactants are adsorbed to the surface of CNTs, but also some of them remain “free” and affect the flow in the evaporating droplet by inducing surface flows. The increasing trend of deposition radius by surfactant addition (which can be seen in Fig. 2b) is also due to the excess of free surfactants. The evaporation-induced flow will carry the CNTs to the contact line and with this flow, some of the surfactants will also be carried to the contact line. The accumulation of the surfactants near the contact line will cause a Marangoni flow due to the gradient in the surface tension at the gas–liquid interface, as shown in Fig. 4. This is the part that makes the physics of the problem even more complicated.^41^ Although adding more surfactant to the CNT colloids before doing all the process of preparation will be helpful for their stability, if the surfactant is added excessively, the remaining free surfactants that are not adsorbed to the CNTs will cause a strong Marangoni flow. This inward Marangoni flow will impede the outward evaporation-induced flow, Marangoni eddies will be created, suppressing the coffee-ring effect; the effect that is desired in our study.

This trend is also perceivable in the ESI videos,† which are taken for a nominal CNT concentration of 0.4 wt%. As the SDS concentration is increased from 0.1 wt% to 0.8 wt%, it is observed that the Marangoni flows (and consequently the eddies) are getting stronger and stronger. Although this addition will make more CNTs stable in the colloid and makes the droplet to be capable of resulting in denser rings after evaporation, the best ring is achieved at the SDS concentration of 0.4 wt%. At the SDS concentration of 0.8 wt% the free surfactants are as much as making strong Marangoni eddies that partially suppress the coffee-ring effect and result in a less dense ring. To offer better insight, the schematic representation of deposited rings and corresponding flows of fluid inside the droplet is depicted in Fig. 4.

By checking Fig. 2, one can notice that for all CNT concentrations, there is a specific SDS concentration at which the ring width is the highest. Thus, there is a critical surfactant concentration at which the colloidal stability is good enough to make a dense CNT ring, but the surfactant-induced Marangoni flows are not strong enough to suppress the ring formation. This critical concentration is called the “optimum” concentration that leads to a kind of “optimal” deposition. In our experiments we found that the optimum surfactant concentration for 0.05 wt%, 0.1 wt%, 0.2 wt%, and 0.4 wt% MWCNT colloids are 0.1 wt%, 0.1 wt%, 0.2 wt%, 0.4 wt% SDS, respectively. As can be seen in Fig. 3, the highest dimensionless ring width and the lowest electrical resistance for each CNT concentration were obtained in these optimum SDS concentrations. Obviously, the results are limited to the chosen step for SDS addition, and it cannot be confidently stated what is the exact SDS concentration for each CNT colloid concentration to get the best results, and more experiments with smaller steps of SDS addition are needed for this matter. This is also the reason that for both suspensions of *ϕ* = 0.05 wt% and *ϕ* = 0.1 wt%, the optimum surfactant concentration is achieved as *ψ* = 0.1 wt% (Fig. 3a). For the suspension of *ϕ* = 0.05 wt%, the optimum SDS concentration may be between 0.05 wt% and 0.1 wt% but closer to the latter. Yet, it can be safely concluded that as the CNT concentration is increased, the SDS concentration at which the best ring is achieved is also increased. As a worth-mentioning point extracted from Fig. 3b, it can be noticed that at *ψ* = 0.1 wt%, CNT concentrations of 0.1 wt% and 0.2 wt% yield more conductive rings than those made by *ϕ* = 0.4 wt%. This is due to the fact that for CNT concentrations of 0.1 wt% and 0.2 wt%, the SDS concentration of 0.1 wt% is optimum or very close to optimum. However, this SDS concentration is quite far from the optimum concentration for *ϕ* = 0.4 wt%. Although the SDS concentration of 0.1 wt% is also the optimum value for *ϕ* = 0.05 wt%, the corresponding rings yield the lowest conductivity due to the lack of enough CNTs for making a well-connected conductive path. Besides, as the CNT concentration increases in optimum SDS concentration, the ratio of *w*/*r* is increased. Similar results were observed by Goh *et al.*^23^ as they examined various concentrations by diluting a stable SWCNT ink. So the optimum dispersant concentration was considered tacitly in their work, and all of their experiments were done in the approximate optimum concentration ratio.

**Fig. 5:**
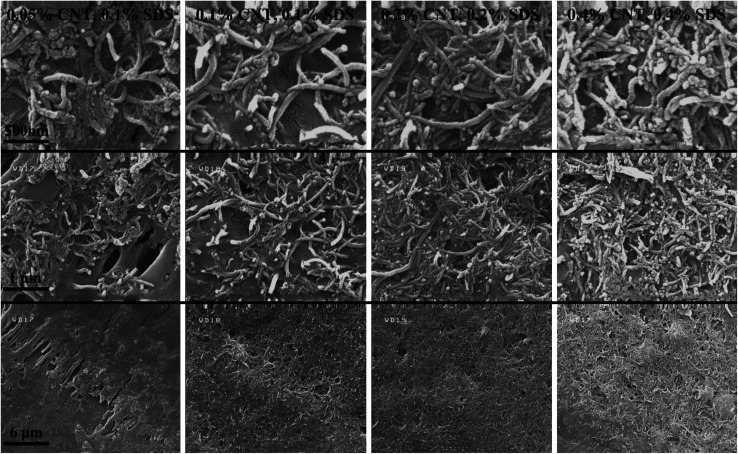
SEM images from the contact line of the final deposition patterns of colloids in different CNT concentrations containing optimum SDS concentration in various magnifications. CNTs seem to be denser and more entangled as their concentration increases, resulting in conductivity enhancement.

SEM images were taken of the contact lines to determine how dense the carbon nanotubes are entangled together. The corresponding images are visible in different magnifications in Fig. 5. It is noticeable that as the CNT concentration is increased, CNTs seem to be more packed together, which is favorable for the overall conductivity of the ring. Consequently, the electrical resistance of the ring deposited from higher CNT concentration is generally lower, as clearly shown in Fig. 3b. It should be noted that although some of the surfactant concentrations were above the apparent critical micelle concentration (CMC) for SDS in water (0.2 wt%), the progressive increase of dispersed CNTs in the colloid as discussed earlier shows that the real CMC is above our highest SDS concentration in this case as this notion was explained in our previous paper.^20^ Succinctly, this is due to the interactions of surfactants with nanoparticles and their adsorption to the surface of the CNTs which are absent in a solution with pure surfactant. As an important point, not passing the CMC leads to a nearly constant viscosity for all suspensions used (listed in Table S3†). It is noteworthy that some astounding patterns are observed in Fig. 2, especially for *ψ* = 0.05 wt%. Their morphology can be characterized by crack formation mechanisms.^42,43^

### Multiple depositions

**Fig. 6:**
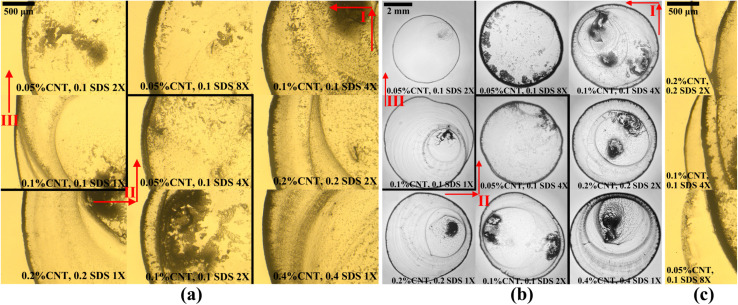
Final deposition patterns of different cases of multiple depositions method in (a) close angle view of successfully achieved rings, (b) full angle view of successfully achieved rings, and (c) close angle view of protruded droplets. The deposits from close view and full view are not necessarily from the same droplet. Images are ordered based on how many times they are printed, and the directions are shown with red arrows. The cases are separated using black lines, and the letter “X” represents the number of times that the deposition is repeated. As the number of depositions increases in each case, the dimensionless ring width also increases.

Finding out from our results that the denser the CNT ring, the better the electrical conductivity, and inspired by previous works that were able to get denser CNT lines by printing them multiple times,^44,45^ we propose the multiple depositions method to get the densest rings possible. Instead of trying CNT colloids with higher concentrations, we tried printing a droplet with lower CNT concentration in the same area multiple times. For this purpose, CNT colloids with optimum SDS concentration were used in order to only compare the best achievable rings. We tried adding the subsequent droplets in different stages of evaporation, *e.g.*, during the pinning mode, at the moment of depinning, and after complete evaporation. Based on our experience, the best way to get better rings and avoid the new droplet protruding was to print the new droplet after the old one was completely evaporated. Fig. 6 shows the results of the ring depositions for different concentrations. For example, it was explored whether a droplet of 0.4 wt% CNT (with 0.4 wt% SDS) will deposit a better ring or depositing droplets of 0.05 wt% CNT (with 0.1 wt% SDS) for 8 times. All the available concentrations in between were also tested and the corresponding results are shown in Fig. 6a(I), Fig. 6a(II) and (III) present similar results for 0.2 wt% CNT and 0.1 wt% CNT. The same results are represented in Fig. 6b, but with images of the whole droplet to give the reader a better insight into the apparent density of the CNT rings for each case.

We also quantified the results by calculating *w*/*r* ratios which are reported in Table 1. Based on the results represented in Fig. 6 and Table 1, it is concluded that the more times a droplet is deposited, the better and denser the ring will be. For example, it is perceivable that printing droplets of 0.05 wt% CNT for 8 times deposits a denser ring than printing droplets of 0.1 wt% CNT for 4 times, 0.2 wt% CNT for 2 times, or 0.4 wt% CNT only for one time. The reason behind this observation lies in the fact that as the first droplet deposits CNTs near the contact line, the ring-shaped deposition constructs a short wall of entangled CNTs on the periphery. By enhancing the pinning force, this wall pins the contact line of the next droplet at the same place, hinders the depinning stage of evaporation, or in other words, enhances the pinning time.^46^ As the pinning mode of evaporation takes longer, the deposited ring will be thicker and denser. Therefore, subsequent printing of the droplets strengthens the wall which is itself the ring deposition – multiple depositions give denser rings than the single deposition. Yet, it was experienced during the experiments that as the number of depositions increased, in spite of using an accurate stage for printing new droplets, the chances of droplets being protruded from the original deposition area increased. There are a few images in Fig. 6c of the protruded droplets. This happens due to the fact that the deposited CNTs may not offer enough resistance against the spreading flow of the next printed droplet. In other words, the wall may not be able to maintain the contact line exactly at the same circular trace.

**Table 1:** The results of the ring width and electrical resistances for different cases of multiple depositions method. The letter “X” represents the number of times that the deposition is repeated. All colloids contain their optimum SDS concentration

Colloid	*w*/*r*	*R* (kΩ)
0.4 wt% CNT 1X	0.034 ± 0.006	24.6 ± 0.1
0.2 wt% CNT 2X	0.047 ± 0.004	22.1 ± 0.1
0.1 wt% CNT 4X	0.045 ± 0.005	13.7 ± 0.1
0.05 wt% CNT 8X	0.068 ± 0.002	8.6 ± 0.1
0.2 wt% CNT 1X	0.024 ± 0.007	40.6 ± 0.2
0.1 wt% CNT 2X	0.029 ± 0.001	25.4 ± 0.1
0.05 wt% CNT 4X	0.043 ± 0.003	15.8 ± 0.2
0.1 wt% CNT 1X	0.018 ± 0.004	90.9 ± 0.2
0.05 wt% CNT 2X	0.036 ± 0.003	66.5 ± 0.1

To get a better idea of the order of magnitudes, it is observable that the case of 8 sequential depositions (*ϕ* = 0.05 wt%, *ψ* = 0.1 wt%) will result in a *w*/*r* ratio which is nearly double the ratio for the one with a single deposition (*ϕ* = *ψ* = 0.4 wt%). The same trend is seen in all the other cases. Similar to the previous part, investigating the level of conductivity of the rings is also important. We carried out a current–voltage analysis for the deposits achieved with the multiple depositions method, and the results are reported in Table 1. As the rings are denser in all cases, the electrical conductivity is also better (that is representative of the fine entanglement of carbon nanotubes). Additionally, the order of errors is much lower since there was a very good connection between the gold probes of the measurement device and the dense enough CNT rings. Similar results were observed by Dinh *et al.*^44^ and Goh *et al.*^45^ for inkjet printing and aerosol jet deposition methods, respectively. As the number of print passes increases, the electrical resistance of CNT twin lines decreases.

### Strain sensing

Carrying out sophisticated strain sensing tests has not been the focus of the present work. Though, as for the proof of concept and ascertaining the fact that this method of depositing CNT rings can be effectively applied in sensing devices, only two groups of tests were carried out. Baring in mind that the densest rings with the highest electrical conductivity were achieved at certain surfactant concentrations, we chose the following colloids for strain sensing: *ϕ* = *ψ* = 0.2 wt% and *ϕ* = *ψ* = 0.4 wt%. For each of these two types, five samples were built and tested. As known well, mechanical strain in a strain gauge is represented by *ε* = Δ*L*/*L* = GF^−1^ × Δ*R*/*R*, where *ε* is strain along the length of a cantilever beam, *L* is length, GF is the gauge factor, and *R* is the electrical resistance of the strain gauge. In a standard Wheatstone bridge circuit with a single variable resistor, relative changes in electrical resistance can be calculated by Δ*R*/*R* = 4 × Δ*V*_o_/*V*_i_, where Δ*V*_o_ is the output voltage difference of the Wheatstone bridge before and after applying strain, and *V*_i_ is the supply voltage of the bridge. By substituting Δ*R*/*R*, we would have *ε* = 4 × GF^−1^ × Δ*V*_o_/*V*_i_. Please note that Δ*R*_contact_ ≪Δ*R*_CNT_ in our tests, so it can be concluded that the total change in electrical resistance (Δ*R* = Δ*R*_CNT_ + 2Δ*R*_contact_) would be approximately equal to Δ*R*_CNT_.

**Fig. 7:**
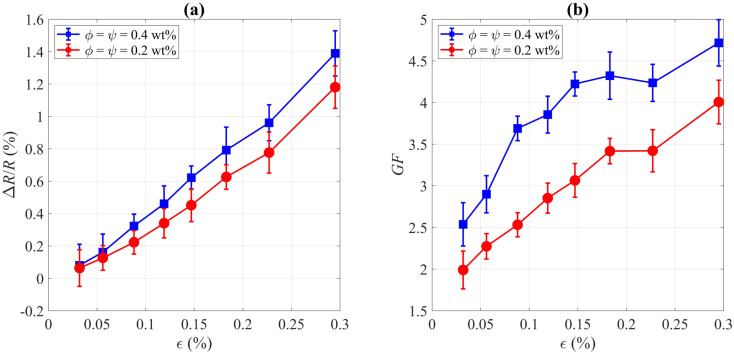
Plots of (a) resistance relative changes and (b) gauge factor measurements *versus* strain for two colloids with different concentrations. Each colloid contains the same concentrations of CNTs (*ϕ*) and SDS (*ψ*). The results indicate gauge factors for the fabricated sensors that are comparable to industrial devices.

For the HBM strain sensor, the gauge factor was 1.97, so we extracted the corresponding strain of each load step on the cantilever beam structure for calibration of the fabricated CNT strain gauges. Having the strain values for each load step, we extracted Δ*R*/*R* and gauge factor for CNT strain gauges, which are represented in Fig. 7. At the maximum strain of 0.3%, the maximum gauge factors were measured 4.0 ± 0.3 and 4.7 ± 0.3 for the colloids with *ϕ* = *ψ* = 0.2 wt% and *ϕ* = *ψ* = 0.4 wt%, respectively, which show significant improvements compared to the reported gauge factors for strain sensors with aligned MWCNTs (GF ≈ 1.4)^47^ or multi-pass printed MWCNT networks (GF ≈ 2.8).^37^ As we observed a linear correlation between the strain and applied force before reaching the yield point of the beam (similar to the linear elastic region of the stress–strain curve), this indicates that the CNTs were not delaminated in our experimental conditions and that sensors work properly in the elastic region. It is worth mentioning that the piezoresistive behavior of CNT rings is potentially induced both by the straining of each individual CNTs which are the load carriers and strain-induced change in the contact area between CNT bundles.^48^

## Conclusions

The findings of the present work suggest that the concentration of surfactant (dispersant) in a CNT colloid has a major impact on the fluid flow inside evaporating colloidal droplets, changing the final deposition patterns. It was demonstrated that for each CNT concentration, there is an optimum surfactant concentration at which the best ring is achieved. Before that specific concentration, there are not enough stabilized dispersed CNTs in the colloid to get dense enough rings. And after that concentration, there would be an excess of free surfactants suppressing the coffee-ring effect by inducing Marangoni eddies and resulting in a poor ring. Based on our experiments, the optimum SDS concentrations as the dispersing agent can be considered to be 0.1 wt%, 0.1 wt%, 0.2 wt%, and 0.4 wt% for colloids with CNT concentrations of 0.05 wt%, 0.1 wt%, 0.2 wt%, and 0.4 wt%, respectively. The electrical resistances for all cases were measured, and it was shown that denser rings have higher electrical conductivity. Therefore, optimum surfactant concentration leads not only to the densest ring deposition but also to the highest electrical conductivity which is due to the high degree of CNTs entanglement as was evident from the SEM images. A new method of multiple depositions was introduced, and it was shown that denser rings with lower resistances can be achieved by this method using lower CNT concentrations. However, there might be more difficulties ahead and more accurate equipment is needed. Lastly, we used the evaporation-driven deposition of CNTs to fabricate simple strain sensors whose gauge factors happened to be higher than the commercial ones, which shows the practicality of the proposed method for industrial applications. The piezoresistive behavior of CNT-reinforced composites has recently been raised for use in autonomous self-healing and structural health monitoring systems.^49,50^

## Author contributions

M. J. I.: formal analysis, investigation, methodology, software, visualization, writing – original draft; J. F.: conceptualization, formal analysis, supervision, visualization, writing – original draft, writing – review & editing; E. A.: methodology, visualization, writing – original draft; S. C.: investigation, writing – original draft; V. B.: conceptualization, funding acquisition, project administration, resources, supervision, writing – review & editing.

## Conflicts of interest

There are no conflicts of interest to declare.

## Supplementary Material

RA-012-D2RA03833A-s001

RA-012-D2RA03833A-s002

RA-012-D2RA03833A-s003

RA-012-D2RA03833A-s004

RA-012-D2RA03833A-s005
