# Supplementary material for: Effect of surfactant concentration on the evaporation-driven deposition of carbon nanotubes: from coffee-ring effect to strain sensing
Source: RSC Adv. 2022 Nov 7;12(49):31688–98. doi: 10.1039/d2ra03833a (PMC9638968; doi:10.1039/d2ra03833a)
Supplement: RA-012-D2RA03833A-s001 [file RA-012-D2RA03833A-s001.pdf]

Electronic Supplementary Material (ESI) for RSC Advances.
This journal is © The Royal Society of Chemistry 2022

Electronic Supplementary Information for:

Effect of surfactant concentration on the evaporation-driven deposition of carbon nanotubes: From coffee-ring effect to strain sensing

Mohammad Jalal Inanlu[1], Jafar Farhadi[1,*], Ehsan Ansari[2], Saina Charkas[1], Vahid Bazargan[1,+]

[1]School of Mechanical Engineering, College of Engineering, University of Tehran, Tehran, Iran

[2]School of Electrical and Computer Engineering, College of Engineering, University of Tehran, Tehran, Iran

[*]jafarfarhadi@ut.ac.ir

[+]vbazargan@ut.ac.ir

1. Concentration of solutions

The true concentration for each solution was measured and the results are represented in Table S1. One solution is used for each set of concentrations, and the value of 5% corresponds to the maximum error in measurement and calculation.

Table S1: The true concentration values (wt% ± 5%) of solutions (**φ:** MWCNT and **ψ:** SDS concentrations in wt%)

φ \ ψ	0.025	0.05	0.1	0.2	0.4	0.8
**0.05**	0.039	0.044	0.047	0.048	--	--
**0.1**	0.077	0.087	0.091	0.094	--	--
**0.2**	0.153	0.166	0.178	0.185	0.191	--
**0.4**	--	--	0.311	0.354	0.365	0.371

2. Surface tension of solutions

To measure the surface tension of the suspensions, first, superhydrophobic surfaces were prepared by etching aluminum samples with hydrogen chloride 2M for 15 minutes and coating them with H,1H,2H,2H-perfluorodecyltriethoxysilane for 4 hours in a furnace at 90°C. Then, five samples of 20 μL drop for each concentration were placed on the surface, and images from the side of the droplets were captured using a CCD camera mounted on the Goniometer (Jikan CAG 20 PE). Using the Jikan Assistant software, the contact angle and curvature fittings data were extracted from the images. Finally, the surface tension data were obtained using the sessile droplet method [1] and shown in Table S2. The results are the mean values with a maximum error of 5%.

Table S2: The surface tension values (mN/m ± 5%) of solutions (**φ:** MWCNT and **ψ:** SDS concentrations in wt%)

φ \ ψ	0.025	0.05	0.1	0.2	0.4	0.8
**0.05**	67.4	59.4	52.9	46.1	--	--
**0.1**	50.2	47.3	42.4	42.3	--	--
**0.2**	49.4	45.9	43.8	43.7	42.6	--
**0.4**	--	--	54.9	44.9	43.3	41.2

3. Viscosity of solutions

Viscosity measurements were carried out using a MODERN Krebs Stormer Viscometer at 100 rpm at 25°C using the viscometer's smallest spindle. The results are listed in Table S3. Since the whole solution was used to measure the viscosity for each concentration, the value of 2% represents the device's accuracy.

Table S3: The viscosity values (mPa.s ± 2%) of solutions (**φ:** MWCNT and **ψ:** SDS concentrations in wt%)

φ \ ψ	**0.025**	**0.05**	**0.1**	**0.2**	**0.4**	**0.8**
**0.05**	0.953	0.955	0.954	0.956	--	--
**0.1**	0.955	0.958	0.961	0.967	--	--
**0.2**	0.965	0.969	0.974	0.981	0.987	--
**0.4**	--	--	0.992	0.998	1.011	1.019

4. References

[1] W. Kwak, J. K. Park, J. Yoon, S. Lee, and W. Hwang, "Measurement of surface tension by sessile drop tensiometer with superoleophobic surface," *Applied Physics Letters* **112**, 121602 (2018).
